# Microbial ecology of the deep terrestrial subsurface

**DOI:** 10.1093/ismejo/wrae091

**Published:** 2024-05-23

**Authors:** Rachel C Beaver, Josh D Neufeld

**Affiliations:** Department of Biology, University of Waterloo, 200 University Avenue West, Waterloo, Ontario N2L 3G1, Canada; Department of Biology, University of Waterloo, 200 University Avenue West, Waterloo, Ontario N2L 3G1, Canada

**Keywords:** deep subsurface, subsurface, groundwater, microbiology, microbial ecology

## Abstract

The terrestrial subsurface hosts microbial communities that, collectively, are predicted to comprise as many microbial cells as global surface soils. Although initially thought to be associated with deposited organic matter, deep subsurface microbial communities are supported by chemolithoautotrophic primary production, with hydrogen serving as an important source of electrons. Despite recent progress, relatively little is known about the deep terrestrial subsurface compared to more commonly studied environments. Understanding the composition of deep terrestrial subsurface microbial communities and the factors that influence them is of importance because of human-associated activities including long-term storage of used nuclear fuel, carbon capture, and storage of hydrogen for use as an energy vector. In addition to identifying deep subsurface microorganisms, recent research focuses on identifying the roles of microorganisms in subsurface communities, as well as elucidating myriad interactions—syntrophic, episymbiotic, and viral—that occur among community members. In recent years, entirely new groups of microorganisms (i.e. candidate phyla radiation bacteria and *Diapherotrites*, *Parvarchaeota*, *Aenigmarchaeota*, *Nanoloarchaeota*, *Nanoarchaeota* archaea) have been discovered in deep terrestrial subsurface environments, suggesting that much remains unknown about this biosphere. This review explores the historical context for deep terrestrial subsurface microbial ecology and highlights recent discoveries that shape current ecological understanding of this poorly explored microbial habitat. Additionally, we highlight the need for multifaceted experimental approaches to observe phenomena such as cryptic cycles, complex interactions, and episymbiosis, which may not be apparent when using single approaches in isolation, but are nonetheless critical to advancing our understanding of this deep biosphere.

## Introduction

The terrestrial subsurface is one of Earth’s largest environments and predicted to host as many microbial cells as global surface soils and more than all oceans combined [1–4]. Especially given the massive volume of this ecosystem, subsurface microbes play an important role in global biogeochemical cycling. The deep terrestrial subsurface is a source of valuable compounds such as ores, minerals, oil, and natural gas. It is also of interest to nuclear waste management organizations for its potential to host deep geological repositories for long-term storage of materials such as used nuclear fuel and other radioactive waste and for its potential in carbon capture and storage of hydrogen for use as an energy vector [5]. Further, certain deep subsurface environments on Earth can serve as analogues to saline subsurface environments on other planets like Mars [6–12]. Nonetheless, the deep terrestrial subsurface remains underexplored, particularly because of logistical challenges of sampling such inaccessible locations.

**Figure 1:**
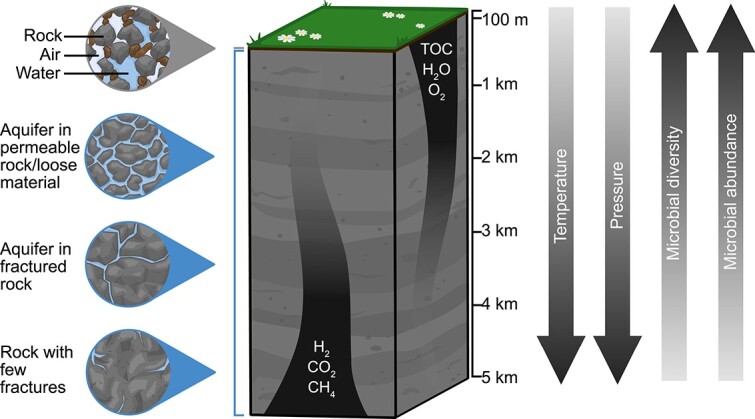
Schematic of a terrestrial subsurface environment. The top layer of the subsurface is generally unsaturated, with saturated layers below. Most aquifers exist in the saturated zone, within the top 100 m from the subsurface. The saturated zone consists of aquifers in permeable rock, loose material, or fractured rock. Outside of aquifers, rock fractures within the saturated zone are often water-filled. As depth increases, so do the temperature and pressure, whereas microbial diversity and abundance are highest closer to the surface. Organic carbon, water, and oxygen become increasingly unavailable with depth, whereas H_2_, CO_2_, and CH_4_ gases are abundant in the deep subsurface.

Microorganisms are diverse in their metabolic needs, but there are several common requirements for all known life on Earth: water, carbon, nutrients, physical space, and energy for growth and reproduction. In many of Earth’s environments, these requirements are met readily, but the deep subsurface is typically nutrient poor. As availability of the necessities of life tends to decrease with depth (Fig. 1), so do the average abundances of microbial cells [13, 14]. In these nutrient-deprived conditions, life in the deep subsurface operates at a slower pace than it does in most surface environments. For example, the average generation time for microbial cells in terrestrial deep subsurface environments has been estimated to be centuries [3, 15–19]. This, coupled with relatively small population sizes, may lead to evolution driven by stochastic processes, like genetic drift, rather than deterministic factors, such as selection [20].

In the deep subsurface, water exists in the form of groundwater, which is a broad term used to describe fluid located below the surface in pore spaces of rocks and soil, in the fractures between rocks, and in aquifers (Fig. 1). Aquifers typically occur in the first 100 m below the surface but can also extend to much greater depths [21]. They are commonly composed of unconsolidated porous rock/sediment (e.g. sand, gravel) or consolidated porous rock (e.g. sandstone). Other aquifers consist of water within interconnected fractures, cracks, or joints in solid rock (Fig. 1). The salinity of groundwater increases with depth and can result in hypersaline environments at some of the greatest depths sampled [22]. Deep groundwater may also host high concentrations of heavy metals, which can be toxic to microorganisms [23, 24].

Given an absence of sunlight, and a lack of associated primary production from photosynthesis, access to organic carbon in the deep subsurface is more limited for microorganisms than it is in surface environments. Some organic carbon in the deep subsurface was included with sediments at the time of their deposition and now through diagenesis exists primarily as oil and petroleum deposits. Subsurface organic carbon also exists in clay, shales, coal, and other deposits. Living near organic carbon deposits can be advantageous for microorganisms, especially heterotrophs, but it is not the only strategy. Organic carbon can also be produced in situ by chemolithoautotrophs that fix inorganic carbon, which allows for microbial life in the subsurface beyond carbon reservoirs [25]. In addition to a lack of organic carbon, deep subsurface environments are often anoxic, and with limited nutrients, thus most subsurface microorganisms rely on non-oxygen electron acceptors and inorganic electron donors for metabolism. However, some deep subsurface environments have access to oxygen via oxidizing water originating from the surface [26]. Alternatively, a recent study demonstrated higher than expected concentrations of dissolved oxygen in old groundwaters that may have been produced *in situ* via microbial dismutation, a process termed “dark oxygen production” [27].

Physical space for microorganisms to inhabit the deep subsurface is highly variable, ranging from pore spaces smaller than the size of a microbial cell to larger fractures and faults that are sometimes interconnected [28]. Rock type influences both pore size and organic carbon availability. Sedimentary rocks are generally more porous than igneous and metamorphic rocks, providing more space for microorganisms to grow and interact [29, 30]. They also generally have not been exposed to the same high-temperature and -pressure conditions as igneous and metamorphic rocks; thus, microbial populations found within them could theoretically have been present since the rock’s deposition [30–32]. In contrast, igneous and metamorphic rocks, which together represent most of the deep subsurface, rely on nutrient and energy source transport via fractures and are usually void of organic matter [30]. Because the pore spaces of these rocks are usually too small for microbial cells, fractures provide the most likely habitats [33, 34].

There is no universal depth that defines the deep terrestrial subsurface biome. Previous publications have described the terrestrial subsurface as deeper than 8 m [3], and the deep terrestrial subsurface as deeper than 100 m [35, 36]. Temperature prevents microbial growth beyond a certain depth [8], increasing by ~25°C per kilometer below the surface in terrestrial environments [34]. This means that any currently known microorganisms could not survive below depths of ~5 km [26]. For the purposes of this review, the deep terrestrial subsurface (also referred to simply as “subsurface” throughout) comprises rocks and groundwater at least 100 m below the surface of continents.

### Historical context

The first documented evidence for subsurface life on Earth was the description of fungi and algae in subterranean gold mines of Guanajuato, Mexico by Alexander von Humboldt in the late 18th century [37]. Despite this early observation, the microbiology of terrestrial environments in general only began with studies of soil in the late 1800s, with researchers initially searching for pathogens. Using the techniques available at the time, Robert Koch first observed that below ~1 m, soil samples were nearly free of bacteria [38]. This conclusion was supported by others studying soil microbiology at the time [39–41], and into the 1900s, where lower numbers of culturable microorganisms, using highly nutritious organic carbon-containing medium, from lower soil depths was attributed to a lack of air and food [42]. Because of early work on soil microbiology that showed very low numbers of microorganisms at the bottom of the soil zone, it was believed that microbial growth below this zone was very limited or non-existent. Coupled with technical challenges sampling the deep subsurface, there was relatively little interest in pursuing the study of deep subsurface microbiology.

Around the 1920s, the presence of hydrogen sulfide in oil reservoirs (“oil souring”) led to predictions that subsurface-associated sulfate-reducing bacteria (SRB) could be responsible. Ernst Georg Wolzogen-Kühr, a German microbiologist, showed the presence of a specific sulfate-reducing bacterium, then referred to as *Microspira desulfuricans*, up to 70 feet below the Earth’s surface [43]. Despite these observations, the geology community believed that sulfate reduction in oil deposits was due entirely to abiotic chemical reactions, and the prevailing opinion remained that the subsurface was sterile. This paradigm was again refuted in a 1926 publication reporting the presence of *Microspira* in crude oil samples from depths of 500 m [44] and again in a 1930 publication [45]. In 1931, Charles Lipman at the University of California, Berkeley presented evidence for microorganisms living in coal samples extracted from 600 m belowground, and he claimed to be the first to postulate that the microorganisms had been there for millions of years, since the deposition of the plant matter that became coal [19]. Over the next few decades, SRB were isolated from several other subsurface oil-well associated environments [46–50]. A dominance of SRB in the literature on subsurface microbiology at this time was likely due to the use of targeted cultivation methods that favored their discovery over other types of microorganisms, as there was interest at the time to confirm their suspected role in oil souring. Nonetheless, additional types of microorganisms were found in oil-deposit samples and other subsurface environments, including *Pseudomonas*, denitrifiers, sulfur oxidizers, and microorganisms capable of using petroleum-associated compounds [51–57]. It was postulated that subsurface soil samples were inhabited by microorganisms with less nutrient adaptability [58], although non-chemoorganoheterotrophic metabolisms were not discussed. Investigations into subsurface microbiology at this time were still largely limited to spring or well water and rarely looked directly at subsurface core material due to difficulties with obtaining such samples.

In the 1970s, agricultural and industrial activities led to groundwater contamination, and one possibility was that subsurface microorganisms could help degrade these contaminants [59]. Several years later, subsurface microbiology gained additional relevance in the context of belowground disposal of radioactive and heavy metal waste. Initial work exploring a potential influence of microorganisms on long-term nuclear waste storage began in the late 1970s in Canada, Switzerland, the UK, and the USA, and soon after in Finland, France, Italy, Japan, and Sweden [60]. By the end of the 20th century, adequate controls and aseptic sampling technique were employed to convince the scientific community that there was indeed microbial life in the subsurface [34].

### Chemical energy for primary production

In the absence of sunlight, subsurface communities must rely on non-photosynthetic primary production. It was originally thought that subsurface life must be supported by organic carbon deposits that were formed by ancient photosynthetic events. Although subsurface microbial communities that are near organic carbon deposits, such as oil, do take advantage of these carbon sources, other communities rely entirely on chemolithoautotrophic metabolism and fix their own carbon from inorganic sources available in the subsurface. The first deep terrestrial subsurface microbial community shown to be completely supported by chemolithoautotrophic primary production was discovered in 1995 [25]. Since then, geogenic gases such as dihydrogen (H_2_), methane (CH_4_), and carbon dioxide (CO_2_) have been linked with belowground primary production [61].

For primary production to occur, microorganisms must have the capacity to fix inorganic carbon into biomass. Several different carbon fixation pathways exist in microorganisms, but perhaps the most important for deep subsurface metabolism is the reductive acetyl-CoA pathway, or Wood–Ljungdahl pathway, because it is the preferred pathway for microorganisms living in low-energy environments near the thermodynamic limit of life [62]. This pathway is commonly used by acetogens, methanogens, and sulfate-reducing microorganisms that, in addition to fixing inorganic carbon, use the pathway for energy production [62]. Metagenomic studies have demonstrated that the reductive acetyl-CoA pathway dominates within deep terrestrial subsurface microbial communities [63–65].

### Hydrogen-driven ecosystems

**Figure 2:**
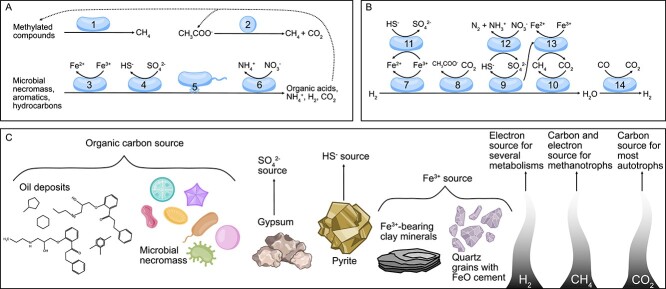
Schematic of deep subsurface microbial metabolisms fueled by organic carbon (A) and H_2_ gas (B), and the sources of electron donors and acceptors in subsurface environments (C). The oxidation of organic carbon compounds can support methylotrophic methanogenesis (1), acetoclastic methanogenesis (2), iron reduction (3), sulfate reduction (4), fermentation, potentially involving episymbiotic relationships (e.g. CPR bacteria and DPANN archaea and their hosts; 5), and nitrate reduction (6). The oxidation of H_2_ gas can support iron reduction (7), acetogenesis (8), sulfate reduction (9), and methanogenesis (10), and the reduced iron, sulfate, and carbon dioxide, and electrons (dashed arrow) produced through these processes can act as electron donors for sulfide oxidation (11), nitrate reduction (12), and anaerobic oxidation of methane (13). Water can be reduced to H_2_, coupled to the oxidation of CO to CO_2_ through the process of carboxydotrophy (14). The metabolisms presented within have been predicted from metagenomic studies [23, 24, 64, 68, 82].

A common feature of deep subsurface microbial communities is a reliance on H_2_ for energy (Fig. 2). Hydrogen gas is present in subsurface environments through processes like radiolysis of water and serpentinization [66]. Although hydrogen-fueled microbial metabolisms in the deep terrestrial subsurface were demonstrated prior to the availability of metagenomics [61], subsequent metagenomic studies have further reinforced a prevalence of genes involved in H_2_ oxidation associated with deep subsurface samples [63–65, 67]. For example, metagenomes generated from samples of three different borehole depths showed a significant enrichment of hydrogenases in borehole samples from 2.3 km compared to those from 0.6 or 1.5 km [24], suggesting that hydrogen becomes increasingly important with distance below the Earth’s surface. Hydrogen gas can be coupled to the reduction of many different electron acceptors that are relevant to deep terrestrial subsurface metabolism, supporting methanogenesis [24, 68–70], homoacetogenesis [24, 71], sulfate/sulfite reduction [16, 65, 68, 72–81], and iron reduction [68, 74, 82–84] (Fig. 2).

### Common microbial subsurface communities

Prior to high-throughput sequencing and metagenomics, deep terrestrial subsurface microbial community characterization generally involved either culturing approaches or clone library analysis (16S rRNA gene amplicon sequencing of selected clones). Using such traditional approaches, deep subsurface communities were commonly reported to be dominated by iron-reducing bacteria, SRB, methanogenic archaea, and acetogens [69, 85–88]. When subsurface samples were taken from locations near hydrocarbon reservoirs, fermentative microorganisms were also detected [89, 90].

Because a subset of microorganisms is favored by cultivation conditions, microbial abundance estimates obtained by these techniques can be much lower than those from microscopy-based techniques, sometimes by orders of magnitude [89]. With the advent of high-throughput amplicon and metagenomic sequencing, it has been possible to study deep biosphere microbial communities with increased resolution, circumventing the biases of culture-based approaches. For additional information about novel techniques for studying deep subsurface environments beyond DNA sequencing–based approaches, see a recent review [91]. A survey of existing amplicon sequencing data from global terrestrial deep subsurface environments showed a universal dominance of phyla *Proteobacteria (Pseudomonadota*) and *Firmicutes* (*Bacillota*). It was proposed that the vast metabolic diversity within these phyla could account for their dominance in deep terrestrial subsurface environments, seemingly independent of underlying geology and environmental factors [36]. Metagenomic studies of deep terrestrial subsurface environments commonly reveal microbial communities with diverse metabolisms. For example, a study of deep subsurface samples from the Horonobe Underground Research Laboratory (Japan) found a diverse microbial community consisting of 29 phyla, including 13 uncultured representatives that had never been detected at this site [68]. The most abundant metabolic functions encoded by the metagenomes were sulfate reduction, sulfur oxidation, nitrate reduction, iron reduction, methane oxidation, and methanogenesis. Almost all reconstructed genomes showed the potential for fermentation, several had genes for nitrogen fixation, many encoded the Calvin–Benson–Basham or reductive acetyl-CoA pathways for carbon fixation, and more than half had genes involved in hydrogen oxidation. The functions detected in these metagenomes are common for deep terrestrial subsurface microorganisms [65, 77, 78, 92–95]; however, not all deep subsurface environments host such diversity (Fig. 2).

### Low-diversity microbial communities

Some deep terrestrial subsurface microbial communities have very low diversity. For example, water circulating within igneous rocks ~200 m belowground in Idaho was sampled and shown to be dominated by methanogens, at >90% of all detected taxa [61]. A similarly low-diversity community was discovered in groundwater from 2.8 km belowground in the Mponeng gold mine in South Africa, which had a microbial community dominated by a single SRB population belonging to the *Firmicutes* (*Bacillota*) phylum [96]. Metagenomic sequencing of fracture fluid recovered from this same environment revealed a metagenome with >99% of reads belonging to this same population’s genome [16]. Additional reads in the metagenome were considered to be laboratory or drilling contaminants. Named *Candidatus* Desulforudis audaxviator, which in Latin means “bold traveler in search of sulfur,” the assembled genome suggested complete self-sufficiency for this subsurface bacterium. In addition to the ability to couple sulfate reduction with H_2_ (e.g. derived from radioactive decay of uranium) or formate oxidation for energy metabolism, the genome for Ca. D. audaxviator contains all genes necessary for carbon and nitrogen fixation and encodes all necessary amino acid biosynthesis pathways. Metabolically flexible, Ca. D. audaxviator can switch from heterotrophy to autotrophy as conditions change. Adaptations such as this could help to explain its ability to thrive in such a harsh environment independently [64]. Since its discovery, Ca. D. audaxviator has been reported in other global subsurface samples [97]. A similarly low-diversity microbial community was later discovered in porous sandstone near an oil deposit, dominated (>98%) by *Halomonas sulfidaeris*, a heterotroph capable of using aromatic organic compounds [23].

### Microeukaryotes

Most research exploring microorganisms in deep terrestrial subsurface environments has focused on bacteria and archaea, but microeukaryotes have been detected as well [98–100]. In bedrock fracture water from Finland, fungi were detected at all tested depths (300–800 m), with the phylum *Ascomycota* being the most prevalent [99]. This study demonstrated a depth-independent distribution of fungal community diversities and several reads associated with potentially novel fungal species. Despite low abundance overall, several fungal species (“mold” and yeast) were detected in groundwater from the Äspo Hard Rock Laboratory [101]. Heat-tolerant taxa from the phylum *Nematoda* have also been detected in subsurface fracture water to depths approaching 3.6 km within the Beatrix gold mine, South Africa [100], where they were suggested to be feeding on prokaryotes. Their heat tolerance may be linked to heat-shock proteins that are transcriptionally induced when these subsurface nematodes grow under heat stress conditions [102, 103]. Additional eukaryotes from phyla *Platyhelminthes*, *Rotifera*, *Annelida*, and *Arthropoda* have been detected in South African mines at approximate depths of 1.5 km belowground [104]. The presence of microeukaryotes in subsurface environments may originate from surface water recharge and, predictably, their subsurface persistence is likely governed by food availability [104, 105].

### Factors influencing subsurface microbial community composition

The factors that affect microbial community composition and diversity of deep terrestrial subsurface environments remain poorly understood. Although the least diverse microbial communities discovered have been in some of the deepest sampled environments [16], other deep subsurface environments host relatively diverse microbial communities [94]. Decreasing diversity with depth is likely a combination of related factors that influence microbial community composition, such as water recharge and origin, water activity (e.g. salinity), organic matter availability, and electron donor and acceptor diversity.

Several 1–5-km-deep samples taken from boreholes in South Africa had microbial communities dominated by either *Firmicutes* (*Bacillota*) or *Proteobacteria* (*Pseudomonadota*) phyla [106–110]. In general, *Proteobacteria* (*Pseudomonadota*) taxa tend to dominate fracture fluids that have more recently mixed with meteoric (i.e. associated with precipitation) waters, which are relatively shallow subsurface fluids. In contrast, representatives of the *Firmicutes* (*Bacillota*) dominate deeper subsurface communities, which tend to be fed from deep groundwaters with little or no meteoric water input [64]. This trend could be explained by the selection for microorganisms, often *Firmicutes* (*Bacillota*) members, capable of using the reductive acetyl-CoA pathway for carbon fixation in lower energy deep environments, with less fluid input from meteoric sources. Indeed, a metagenomics study observed a higher relative abundance of *Firmicutes* (*Bacillota*) members in fracture fluids with little mixing of meteoric waters, which was associated with a higher abundance of protein-encoding genes associated with the reductive acetyl-CoA pathway [64]. A correlation between water origin and microbial community composition has been reported for other environments, including the Fennoscandian Shield and serpentinite springs in Canada [111, 112]. Water recharge, as well as organic matter availability, is also reported to be positively correlated with subsurface microbial community diversity [64]. Although addressed by these experiments, additional factors could favor the persistence of certain microorganisms at greater depths compared to others, such as the ability of some microorganisms, including members of the *Bacillota*, to form spores and withstand unfavorable conditions.

In addition to carbon fixation pathways, other adaptations for nutrient-poor conditions of the deep subsurface could help explain persistence of certain microorganisms in these environments. For example, *H. sulfidaeris*, which was found to dominate (>98%) a microbial community in sandstone, is well adapted to use the various aromatic organic compounds available nearby due to oil deposit proximity [23]. It also has adaptations for survival in the hypersaline subsurface, including transmembrane transporters for ions, heavy metal and ion efflux pumps, and various other osmotic regulators. As a facultative anaerobe, it can also adapt to changes in oxygen availability and is tolerant to high temperature and pressure [23]. The microorganisms detected at the deepest depth sampled in a borehole in Finland had similar adaptations to the high salt and metal concentrations [24]. Some obligate fermenting microorganisms can use the osmoprotectant compounds produced by other organisms as a carbon and energy source. It was observed that the microbial community composition in 2.5-km-deep shale wells in Pennsylvania shifted in response to increasing salt concentration associated with hydraulic fracturing of shale to favor halotolerant bacterial and archaeal species: *Candidatus* Frackibacter, which was discovered at the site, *Halanaerobium*, *Halomonadaceae*, *Marinobacter*, *Methanohalophilus*, and *Methanolobus* [113]. All genomes had evidence of an osmoprotectant strategy, including use of the molecule glycine betaine, proposed to be produced by other microorganisms present to fuel their fermentative metabolisms [113]. Another proposed adaptation to oligotrophic deep subsurface conditions is small cell size [114]. Approximately 50% of the cells in microbial communities of groundwater collected from the Äspö Hard Rock Laboratory passed through a 0.22-μm filter. These small cells often had genomes that were assigned to phylum *Proteobacteria* (*Pseudomonadota*), and all had matches to known representative species reported to have cell sizes larger than 0.3 μm [114].

Another factor that has been shown to influence microbial community composition is the underlying geology of deep terrestrial subsurface environments. Microorganisms often make use of the molecules and ions available in the rocks they inhabit, either as electron sources or as sources of limiting minerals (Fig. 2). This includes metal sulfides like pyrite [115, 116], metals such as iron and manganese and their oxides [117], silicate rocks like feldspar that provide a source of phosphorus [118, 119], and gypsum-derived sulfate [68], which are not evenly distributed in all rocks [116]. Profiles of available electron donors in subsurface ecosystems correlate with microbial community composition [120], but host rock lithology has rarely been directly linked to microorganisms living within that rock. Nonetheless, one study compared the lithology and microbial community compositions of 15 types of host rock taken from many different locations and showed that host rock lithology was a primary driver of microbial community structure [36]. A study out of the Deep Mine Microbial Observatory (South Dakota) looking at biofilms in fluid-filled fractures supports these results and suggests that the types of minerals present could be an important factor for which microorganisms colonize the rock surfaces [121]. Similarly, microbial communities within granite were dependent on mineral inclusions, especially those containing aluminum, silica, and calcium [122]. Another study showed that aquifer fluid type (e.g. gabbro, hyperalkaline peridotite, and alkaline peridotite) was correlated to microbial community composition [123]. Although a single geochemical parameter accounted for the correlation, differing pH, Eh, and availability of carbon and electron acceptors among rock types were predicted to be key factors [123]. As microorganisms use the minerals present in the rock, they chemically transform them. While this process has been studied in surface environments such as clay minerals in soil [124, 125], it is an important consideration to make in deep subsurface environments, especially when they will be modified and potentially amended with non-native materials (e.g. clay, concrete) through the construction of underground repositories, such as for long-term storage of used nuclear fuel, carbon capture, and hydrogen storage.

A recent study showed that stochastic geological activity may play a role in microbial community structure and succession, with a stronger influence than environmental selection for deep hard rock aquifer systems [126]. The findings suggest that geological activity causing or changing fractures, which leads to the isolation or mixing of fracture fluids and the nutrients and microbial communities within, plays a significant role in microbial community turnover and the establishment of new microbial communities when environmental conditions and underlying geology of the rock formation remain unchanged [126]. Further understanding the factors that determine microbial community composition and drive succession in deep terrestrial subsurface environments will be critical for the planning of deep subsurface activities that could be impacted by microbial activity, such as the construction of underground repositories for used nuclear fuel storage.

### Ecological interactions within the subsurface

#### Biofilms

As is the case in most environments, many deep terrestrial subsurface microorganisms exist in biofilms. The proximity of different groups of biofilm microorganisms makes many of the interactions discussed below possible. In the deep subsurface, biofilms can form on rock fractures and in pore spaces, which are very poorly studied compared to deep subsurface fluids like groundwater due to the difficulty of obtaining such samples [121]**.** Biofilms have been shown to be naturally present on rock fractures [75, 127–130], and their microbial community composition differs from surrounding groundwater [75, 128]. Initial studies on deep subsurface biofilm have shown that mineral composition of the rock plays a role in biofilm formation, size, and composition [121, 130]. Deep subsurface biofilms could be an important environment for continued study to build our understanding of microbial interactions in the deep subsurface.

#### Interconnectedness of microbial metabolisms

Most studied deep terrestrial subsurface environments have microbial community members capable of metabolic processes that are often interdependent. Metabolic end products from one population can be used as electron sources for another (Fig. 2). For example, interspecies hydrogen transfer is a key interaction that has been observed or suggested for various anoxic environments. This process can reduce the partial pressure of hydrogen in the immediate environment sufficiently for H_2_-producing metabolic reactions such as acetogenesis to become thermodynamically favorable [131]. Within the subsurface environment context, a simple community consisting of *Pseudomonas* and a SRB belonging to the family *Peptococcaceae* was discovered in Opalinus Clay borehole water via metagenomic sequencing [78]. It was proposed that *Pseudomonas* ferments organic macromolecules, potentially leached from the clay, which releases organic acids and H_2_ gas. In turn, the SRB population couples organic acid oxidation to sulfate reduction. In fermentative communities, sequential fermentation steps performed by multiple different syntrophs can prevent the build-up of fermentation products. Although the roles of anaerobic fungi in deep subsurface environments are poorly understood, the discovery of fossilized fungi in deep anoxic fractured crystalline water suggest that they may also be involved with interspecies hydrogen transfer in deep terrestrial subsurface systems, similar to their well-studied rumen counterparts [132].

In some cases, microorganisms with “complementary” metabolisms living in close association with one another results in cryptic cycles that can make it challenging to detect metabolic activity because the concentrations of electron acceptors and donors remain low despite active cycling. With sulfur in particular, this can have the added advantage of preventing the accumulation of toxic end products; sulfide produced by SRB does not reach toxic concentrations when it is rapidly depleted by sulfide oxidizers. Evidence for such cryptic sulfur cycling in the subsurface includes metagenomic sequencing of deep subsurface sediments from the Horonobe Underground Research Laboratory, which revealed a high relative abundance of microorganisms capable of sulfur cycling, despite consistently low concentrations of sulfate and sulfide in the associated groundwater [68]. A similar observation was made for groundwater from ~300 m belowground in Sweden, where there was undetectable sulfide in the water, but sulfate-reducing and sulfide-oxidizing bacteria were both abundant in the metagenomes, further suggesting that cryptic sulfur cycling could be occurring [65]. Results such as these highlight the importance of combining multiple experimental techniques to study these poorly understood ecosystems.

Another less-well understood form of syntrophy in deep terrestrial subsurface environments is the sharing of electrons between anaerobic methanotrophic (ANME) archaea and other groups of microorganisms, such as sulfate-reducing bacteria, which has been suggested to occur directly via a nanowire structure rather than through the exchange of electron donors [133]. In subsurface environments where both ANME archaea and methanogens are present, a cryptic carbon cycle can exist where methane is produced by the methanogen, and used by the methanotroph, which, in turn, produces carbon dioxide that can be used by the methanogen [68].

Microorganisms with interconnected metabolisms may be even more prevalent in subsurface environments than currently recognized. A recent metagenomics study suggested that most microorganisms within subsurface groundwater communities were incapable of performing multiple sequential redox transformations, including complete sulfide oxidation to sulfate and complete denitrification to N_2_ gas, and instead, the pathways were performed through multiple different species living in close association with one another [134]. Although metagenomics can provide predictions about potential interactions, future studies will need to couple metagenomics with techniques such as enrichment cultivation, microscopy, and isotope labeling techniques to demonstrate such syntrophic relationships.

#### Episymbiosis

The recent discovery of the candidate phyla radiation (CPR) of bacteria as well as DPANN (an acronym of the first five phyla included in the superphylum: *Diapherotrites*, *Parvarchaeota*, *Aenigmarchaeota*, *Nanoloarchaeota*, *Nanoarchaeota*) archaea in deep terrestrial subsurface environments suggests an important role for episymbiosis in deep subsurface environments. Both CPR bacteria and DPANN archaea are relatively abundant in groundwater, and are generally episymbiotic, attaching to host cells [135]. Studies also show that CPR bacteria can be detected in some of the deepest sampled environments [136, 137]. For the DPANN archaea, metagenomes obtained from several years of samples from a deep aquifer system [138] demonstrate consistent co-occurrence patterns for a DPANN symbiont, *Candidatus* Huberiarchaeum crystalense, and its host, *Candidatus* Altiarchaeum hamiconexum, with several characteristics similar to the well-studied relationship between *Nanoarchaeum equitans* (also DPANN) and its host *Ignicoccus hospitalis*. Although the presence of Ca. H. crystalense and its host has not been reported for many deep subsurface environments, likely due to their recent discovery, it may well be that they are difficult to detect using traditional sampling methods due to their small size (i.e. passing through sample filters), unusual ribosome structure, and missing ribosomal proteins [139]. The metabolic and ecological roles of CPR and DPANN are not yet well known, but many members possess genes for fermenting carbon compounds to produce acetate, lactate, formate, and ethanol, possibly using polysulfides as terminal electron acceptors [140] (Fig. 2). Other studies suggest that some episymbiotic taxa could play metabolic supporting roles in nitrite reduction to ammonia and sulfate reduction [135]. Both CPR and DPANN representatives likely benefit from their hosts by scavenging vitamins, sugars, nucleotides, and reduced redox equivalents [138], as well as membrane lipids [141]. Others have speculated that S-layer production by several of these episymbionts could play a protective role against viruses for host cells [142–144]. Additional metagenomics studies of deep subsurface environments are necessary to develop an improved understanding of the impact of DPANN and CPR members on microbial community ecology and biogeochemical cycling within the deep subsurface.

#### Viruses

It has long been known that viruses play an important role in driving microbial diversification and controlling the balance of microbial communities in well-studied environments. Until recently, little was known about the role of viruses in deep terrestrial subsurface environments. To first determine if viruses were present in the deep subsurface, granitic groundwater samples from 69- to 450-m deep in the Äspö Hard Rock Laboratory (Sweden) were analyzed [145]. Overall, cell abundances and viral counts indicated that viruses from seven different families, including several known lytic viruses, were present and were about 10-fold more abundant than bacterial and archaeal cells. This suggests that viruses have a similarly important role in controlling the abundance of subsurface microbial populations as they do in more well-characterized aquatic, terrestrial, and host-associated environments. A single-celled genomics approach showed evidence for viral infection of a *Firmicutes* (*Bacillota*)-dominated community in fracture water from 3-km deep in South Africa [146] and a recent study discovered two new bacteriophages native to groundwater [147], together suggesting that subsurface environments host diverse and yet to be discovered populations of bacteriophages.

## Conclusions

Deep terrestrial subsurface microbiology is still a relatively new field, with immense opportunity for further exploration and discovery. The widespread availability of metagenomic techniques has allowed researchers to explore subsurface microbial communities at a resolution not previously possible and has offered insight into the metabolisms and adaptations that these microorganisms use to survive relatively harsh conditions deep below the Earth’s surface. Although metagenomics can generate hypotheses about metabolic roles and symbiotic interactions, future research involving enrichment cultivation and microcosm experiments should ideally be coupled to cultivation-independent techniques. Together, these approaches can demonstrate how subsurface microorganisms interact with one other and confirm that taxa detected *in situ* represent living and viable microorganisms rather than relic DNA, for example. For future microbial ecology studies of the subsurface, an important goal will continue to be elucidating factors that govern microbial distributions, as well as the factors that influence deep subsurface microbial community diversity. Research is still leading to the discovery of new types of microorganisms, such as CPR bacteria and DPANN archaea, that evaded detection using traditional characterization methods. These recent findings suggest that we are just scratching the surface of belowground microbial diversity. Sampling of deep subsurface environments remains challenging and has largely been limited to mines and boreholes that are constructed for reasons aside from microbiology. Our understanding of the deep terrestrial subsurface is limited to these “windows” of sampling opportunity, and there remain vast expanses of the deep subsurface that are completely unexplored.

Various studies on deep subsurface microbiology to date have given us a perspective of what is happening, but it remains challenging to make broad generalizations of subsurface life because it is unclear how generalizable observations from individual sites might on a global scale. Increased understanding of the microorganisms capable of living in deep terrestrial subsurface environments, and the factors that influence their growth, will help with modelling global biogeochemical cycling and making predictions about future subsurface activities in relation to human activities such as mining and nuclear waste storage.
